# Optimizing high-flow nasal cannula flow settings in adult hypoxemic patients based on peak inspiratory flow during tidal breathing

**DOI:** 10.1186/s13613-021-00949-8

**Published:** 2021-11-27

**Authors:** Jie Li, J. Brady Scott, James B. Fink, Brooke Reed, Oriol Roca, Rajiv Dhand

**Affiliations:** 1 Division of Respiratory Care, Department of Cardiopulmonary Sciences, Rush University, 600 S Paulina St, Suite 765, Chicago, IL 60612 USA; 2 Aerogen Pharma Corp, San Mateo, CA USA; 3 Servei de Medicina Intensiva, Hospital Universitari Vall d’Hebron, Barcelona, Spain; 4 Ciber Enfermedades Respiratorias (Ciberes), Instituto de Salud Carlos III, Madrid, Spain; 5 Division of Pulmonary and Critical Care Medicine, Department of Medicine, University of Tennessee Graduate School of Medicine, Knoxville, TN USA

**Keywords:** High-flow nasal cannula, Flow setting, Peak inspiratory flow, Hypoxemia

## Abstract

**Background:**

Optimal flow settings during high-flow nasal cannula (HFNC) therapy are unknown. We investigated the optimal flow settings during HFNC therapy based on breathing pattern and tidal inspiratory flows in patients with acute hypoxemic respiratory failure (AHRF).

**Methods:**

We conducted a prospective clinical study in adult hypoxemic patients treated by HFNC with a fraction of inspired oxygen (F_I_O_2_) ≥ 0.4. Patient’s peak tidal inspiratory flow (PTIF) was measured and HFNC flows were set to match individual PTIF and then increased by 10 L/min every 5–10 min up to 60 L/min. F_I_O_2_ was titrated to maintain pulse oximetry (SpO_2_) of 90–97%. SpO_2_/F_I_O_2_, respiratory rate (RR), ROX index [(SpO_2_/F_I_O_2_)/RR], and patient comfort were recorded after 5–10 min on each setting. We also conducted an in vitro study to explore the relationship between the HFNC flows and the tracheal F_I_O_2_, peak inspiratory and expiratory pressures.

**Results:**

Forty-nine patients aged 58.0 (SD 14.1) years were enrolled. At enrollment, HFNC flow was set at 45 (38, 50) L/min, with an F_I_O_2_ at 0.62 (0.16) to obtain an SpO_2_/F_I_O_2_ of 160 (40). Mean PTIF was 34 (9) L/min. An increase in HFNC flows up to two times of the individual patient’s PTIF, incrementally improved oxygenation but the ROX index plateaued with HFNC flows of 1.34–1.67 times the individual PTIF. In the in vitro study, when the HFNC flow was set higher than PTIF, tracheal peak inspiratory and expiratory pressures increased as HFNC flow increased but the F_I_O_2_ did not change.

**Conclusion:**

Mean PTIF values in most patients with AHRF were between 30 and 40 L/min. We observed improvement in oxygenation with HFNC flows set above patient PTIF. Thus, a pragmatic approach to set optimal flows in patients with AHRF would be to initiate HFNC flow at 40 L/min and titrate the flow based on improvement in ROX index and patient tolerance.

*Trial registration*: ClinicalTrials.gov (NCT03738345). Registered on November 13th, 2018. https://clinicaltrials.gov/ct2/show/NCT03738345?term=NCT03738345&draw=2&rank=1

**Supplementary Information:**

The online version contains supplementary material available at 10.1186/s13613-021-00949-8.

## Introduction

During high-flow nasal cannula (HFNC) therapy, oxygenation is improved by delivering supplemental oxygen at a flow that exceeds the patient’s peak inspiratory flow [1, 2]. Numerous randomized controlled trials and meta-analyses have shown that HFNC improves oxygenation and reduces the need for intubation in hypoxemic patients compared with conventional oxygen therapy [3–8]. Additionally, a recent clinical practice guideline provides a strong recommendation for use of HFNC in patients with acute hypoxemic respiratory failure (AHRF) [6].

Flow settings play a critical role when using HFNC, as the physiological effects of HFNC are flow-dependent. The higher the flow, the greater is the improvement in inspiratory effort and dynamic lung compliance [9–12]. This has led many clinicians to arbitrarily initiate HFNC at the higher flow settings, such as 60 L/min for adults; however, higher flows may not be well tolerated or associated with optimal oxygenation in all patients [13]. When the HFNC flow is set to match or exceed patient peak tidal inspiratory flow (PTIF), positive end-expiratory pressure (PEEP) begins to be generated [14], and a linear increase in PEEP occurs with an incremental increase in gas flow while breathing with the mouth closed [14–17]. In vitro studies have reported that the measured fraction of inspired oxygen (F_I_O_2_) at the nose level is lower than the set F_I_O_2_ when HFNC flow is set lower than PTIF [18].

The PTIF in adults varies greatly by disease and may be high in the presence of respiratory distress [17, 19–21]. HFNC flows used in many studies and real-life clinical practice also vary widely, from 20 to 60 L/min [22]. Moreover, breathing patterns, including PTIF, tidal volume (Vt), inspiratory time (Ti), and respiratory rate (RR), of patients with AHRF who are treated by HFNC have not been well characterized and there is little guidance on optimal HFNC flow settings based on patient’s breathing patterns.

Therefore, in this clinical study, we studied breathing patterns of patients with AHRF treated by HFNC and measured their PTIF. We also assessed patient clinical response and changes in comfort with different HFNC flows that matched or exceeded the measured PTIF. We hypothesized that patient oxygenation would improve as the ratio of HFNC gas flow to PTIF increased. Finally, we performed an in vitro study that used the breathing patterns acquired from the clinical study to analyze tracheal F_I_O_2_ and airway pressures changes that are associated with different HFNC flow settings.

## Methods

### Clinical study

This prospective observational study was approved by the ethics committee (Approval No. 18102503-IRB01) and implemented in adult ICUs at Rush University Medical Center. Due to the noninvasive features of the study, written consent was waived by the ethics committee, and verbal approval was acquired from patients. The study was registered with ClinicalTrials.gov (NCT03738345).

#### Study population

Adult patients aged 18–90 years who were receiving HFNC and required a minimum F_I_O_2_ of 0.4 to maintain pulse oximetry (SpO_2_) of 90–97% were included. Patients were excluded if they met any of the following criteria: pregnant, non-English speaking, unable to verbally communicate, or hemodynamically unstable. Patients who received inhaled pulmonary vasodilator via HFNC, were receiving extracorporeal membrane oxygenation (ECMO), or were unable to use a mask (facial trauma or claustrophobia) were also excluded.

#### Study procedures

Eligible patients were approached by study investigators to thoroughly explain the study, using a written information sheet. After giving verbal approval, patients were disconnected from HFNC and placed on a properly fitting full-face mask (Airlife mask, Carefusion, San Diego, USA), which was connected to a flow sensor and a Y-piece. The flow sensor was connected to a monitor (NICO2, Respironics, Murrysville, USA) to measure patient PTIF, Ti, RR, and Vt. The Y-piece was attached to two one-way valves, with one that allowed exhalation and the other enabled inhalation from a reservoir bag, which was connected to a back pressure compensated flowmeter from an oxygen-air blender (Additional file 1: Fig. S1). Blender gas flow was adjusted to maintain reservoir bag inflation of 1/2 to 3/4 full while patients were breathing, with F_I_O_2_ titrated to maintain SpO_2_ of 90–97%. This setup allowed patients to breathe fresh gas with a constant F_I_O_2_ during measurement to truly reflect their breathing profiles at a constant F_I_O_2_. Patients were instructed to breathe normally with the mask for at least 2 min. Breathing profiles were recorded when patient breathing appeared to be stable. A minimum of five breathing cycles were recorded and average values of PTIF, Ti, RR, and Vt were calculated.

Once breathing parameters were acquired, patients were returned to HFNC with the previous flow setting if it was lower than PTIF or with flow set at the PTIF level. Then the HFNC flow was progressively increased by 10 L/min every 5–10 min up to 60 L/min or the highest flow the patient could tolerate. At each flow setting, F_I_O_2_ was titrated to maintain SpO_2_ of 90–97%.

#### Outcome

The primary outcome was the SpO_2_/F_I_O_2_ at different HFNC flows above PTIF compared to the SpO_2_/F_I_O_2_ at HFNC flow that matched patient PTIF (defined as matching flow). The secondary outcome included RR, ROX index [(SpO_2_/F_I_O_2_)/RR] [23], and patient comfort scores at different flows. Comfort was self-reported by each patient using a visual numerical scale with a score of 0 as the least and 10 as the most comfort. [13, 21].

#### Sample size

This study was a single group pre–post comparison study designed to compare the change of SpO_2_/F_I_O_2_ ratio with changes in HFNC flow. Using a mean SpO_2_/F_I_O_2_ of 200, a standard deviation of 50 [11, 12] and SpO_2_/F_I_O_2_ increase of 10% to calculate the sample size, with confidence level (1 − α) of 95% and power (1 − ß) of 80%, the number of patients was 49.

### In vitro study

#### Experiment setup

An adult manikin (Laerdal adult airway management trainer, Stavanger, Norway) with size-appropriate airway anatomy was attached to one chamber of a model lung (TTL, Michigan Instruments, Grand Rapids, USA), while the other chamber was connected to a critical care ventilator (Drager Evita XL, Drager, Lubeck, Germany) to simulate respiratory drive. The two chambers moved together via a rigid metal connector to simulate spontaneous breathing. Ventilator settings were adjusted to replicate the breathing patterns that were acquired from patients, along with their flow settings in the clinical study, and breathing patterns were confirmed by NICO2 monitor. Between the trachea and the model lung, a pressure manometer and an oxygen analyzer were connected via a T-piece to measure F_I_O_2_ and pressure, respectively (Additional file 1: Fig. S2). The manikin’s mouth was taped to simulate nose-breathing, and a nasal cannula of large-size was attached to the nose with dry gas administered from blender and flowmeter. Tracheal peak inspiratory and expiratory pressure and F_I_O_2_ were recorded after a minimum of 2 min at each flow setting.

### Statistical analysis

The Kolmogorov–Smirnov test was used to test the normality of distribution for considered variables. Continuous variables among different flows were expressed as mean [standard deviation (SD)] or median [inter-quartile range (IQR)] based on the distribution of variables. Repeated measures ANOVA or Friedman test was used to compare the differences among continuous variables at different flows, while post hoc correction for all pair-wise multiple comparisons were performed using Bonferroni method. Pearson or Spearman correlation analysis was conducted to explore the correlation. *p* < 0.05 was considered statistically significant for all tests. Data analysis was conducted with SPSS software (SPSS 23.0; Chicago, IL).

## Results

### Clinical study

From December 26th, 2018, to March 30th, 2021, 49 hypoxemic patients treated by HFNC were recruited. Thirty-three (67%) patients had pulmonary etiology of AHRF. At the time of study enrollment, HFNC gas flow was set at 45 (38, 50) L/min, with F_I_O_2_ at 0.62 (0.16). SpO_2_/F_I_O_2_ was 160 (40) and ROX index was 7.65 (3.05) (Table 1).

**Table 1:** Patients’ characteristics

	Overall
No. of patients	49
Age, mean (SD), years	58.0 (14.1)
Gender (male), *n* (%)	27 (55%)
Height, mean (SD), cm	167.7 (10.5)
Predicted body weight, mean (SD), kg	61.9 (11.2)
Ethnicity, *n* (%)	
African American	18 (37%)
Caucasian	17 (35%)
Hispanic/Latino	10 (20%)
Asian	4 (8%)
Smoker, *n* (%)	21 (43%)
Smoking package years	20 (3.5, 39)
Cause of acute hypoxemic respiratory failure	
COVID-19 pneumonia	25 (51%)
Postoperative respiratory failure	10 (20%)
Non-COVID-19 pneumonia	5 (10%)
Congestive heart failure	5 (10%)
Lung cancer	3 (6%)
Sickle cell anemia, acute chest	1 (2%)
Comorbidity, *n* (%)	
Chronic lung disease	8 (16%)
Chronic heart disease	17 (35%)
Hypertension	29 (59%)
Diabetes mellitus	13 (27%)
Obesity (BMI ≥ 30 kg/m^2^)	9 (18%)
Obstructive sleep apnea	7 (14%)
Cancer	15 (31%)
HFNC parameters at study enrollment	
F_I_O_2_, mean (SD)	0.62 (0.16)
SpO_2_, median (IQR), %	94 (93, 95)
SpO_2_/F_I_O_2_, mean (SD)	160 (40)
ROX index, mean (SD)	7.65 (3.05)
Gas flow, median (IQR), L/min	45 (37.5, 50)
Breathing measurement	
Tidal volume, median (IQR), ml	468 (399, 548)
RR, median (IQR), bpm	21 (18, 26)
Ti, mean (SD), sec	1.24 (0.41)
Peak tidal inspiratory flow, mean (SD), L/min	34 (9)
Peak tidal inspiratory flow, median (IQR), L/min	31 (27, 42)

HFNC, high-flow nasal cannula; F_I_O_2_, fraction of inspired oxygen; SpO_2_, pulse oximetry; RR, respiratory rate; ROX, (SpO_2_/ F_I_O_2_)/RR; Ti, inspiratory time; BMI, body mass index; SD, standard deviation; IQR, inter-quartile range

#### Breathing patterns

Breathing patterns for the 49 patients were: Vt of 468 (399–548) mL, RR of 21 (18–26) breaths/min, Ti of 1.24 (0.41) second, and PTIF of 34 (9) L/min (Table 1). There was no observed correlation between PTIF and SpO_2_/F_I_O_2_ (*r* = 0.094, *p* = 0.52) or ROX index (*r* = − 0.174, *p* = 0.23).

#### Patient responses to flows above their matching flow

Among the 49 patients, PTIFs close to 20, 30, 40, and 50 L/min were recorded in 4 (8%), 27 (55%), 14 (29%) and 4 (8%) patients, respectively. All patients, except one whose matching flow was 30 L/min, tolerated the maximum gas flow of 60 L/min. As such, only 30 patients received HFNC flows of 10, 20, and 30 L/min above their matching flow. Among the 30 patients, compared to SpO_2_/F_I_O_2_ at the matching flow, SpO_2_/F_I_O_2_ was higher with HFNC flow set 10 L/min (*p* < 0.001) and 20 L/min (*p* < 0.001) higher than the matching flow (Additional file 1: Fig. S3 and Table 2), no further improvement was observed 30 L/min above matching flow. Similarly, ROX index was significantly improved at 10 L/min (*p* = 0.015) and 20 L/min (*p* < 0.001) above matching flow but there was no further increase at 30 L/min above matching flow. RR and comfort scores were not significantly different among different flows. Similar responses were also found among the 14 patients who had mean PTIF of 40 L/min and only received HFNC flows of 10 and 20 L/min above matching flow (Additional file 1: Fig. S4 and Table 2). In these patients a significant reduction of RR with 20 L/min above matching flow was also observed.

**Table 2:** Patient responses to HFNC flows set above their matching flow

PTIF	Parameters	At matching flow	At 10 L/min above matching flow	At 20 L/min above matching flow	At 30 L/min above matching flow	*p*
20–30 L/min (*n* = 30)	SpO_2_, %	93.8 (1.5)	93.4 (1.6)	93.5 (1.5)	93.2 (1.8)	0.151
	F_I_O_2_	0.63 (0.45, 0.82)	0.60 (0.42, 0.71)*^,†^	0.54 (0.40, 0.62)*^,†^	0.49 (0.36, 0.60)*^,†,&^	< 0.001
	SpO_2_/F_I_O_2_	161.3 (50.9)	181.1 (61.2)*	200.5 (66.9)*^,†^	207.1 (69.7)*^,†^	< 0.001
	No. of patients with SpO_2_/F_I_O_2_ improvement compared to the previous flow, %	NA	27 (90%)	26 (87%)	17 (57%)	NA
	No. of patients whose SpO_2_/F_I_O_2_ improvement ≥ 20% compared to baseline, %	NA	8 (27%)	18 (60%)	19 (63%)	NA
	RR, bpm	23.4 (6.5)	23.2 (6.5)	22.2 (5.8)	22.5 (5.6)	0.093
	ROX index	7.5 (3.2)	8.5 (4.0)*	9.8 (4.7)*^,†^	10.0 (4.7)*^,†^	< 0.001
	Comfort	8.0 (5.8, 9.6)	8.0 (7.0, 9.5)	8.0 (5.0, 9.3)	8.0 (5.3, 10.0)	0.712
40 L/min (*n* = 14)	SpO_2_, %	93.2 (2.0)	93.1 (2.1)	92.9 (2.0)	NA	0.623
	F_I_O_2_	0.52(0.45, 0.64)	0.48 (0.42, 0.60)*	0.45 (0.40, 0.58)*^,†^	NA	< 0.001
	SpO_2_/F_I_O_2_	172.0 (40.3)	186.5 (44.2)*	195.9 (41.7)*^,†^	NA	< 0.001
	No. of patients with SpO_2_/F_I_O_2_ improvement compared to the previous flow, %	NA	11 (79%)	10 (71%)	NA	NA
	No. of patients whose SpO_2_/F_I_O_2_ improvement ≥ 20% compared to baseline, %	NA	1 (7%)	4 (29%)	NA	NA
	RR, bpm	27.9 (10.8)	26.3 (11.9)	25.6 (10.5)*	NA	0.016
	ROX index	6.9 (2.7)	8.4 (3.7)*	9.0 (4.2)*	NA	0.006
	Comfort	8.0 (7.0, 8.3)	8.0 (6.8, 9.6)	7.5 (5.0, 9.0)	NA	0.607
50 L/min (*n* = 4)	SpO_2_, %	93.0 (2.2)	93.0 (1.4)	NA	NA	0.713
	F_I_O_2_	0.64 (0.19)	0.57 (0.16)	NA	NA	0.066
	SpO_2_/F_I_O_2_	152.7 (35.3)	170.7 (39.6)	NA	NA	0.068
	No. of patients with SpO_2_/F_I_O_2_ improvement compared to the previous flow, %	NA	4 (100%)	NA	NA	NA
	No. of patients whose SpO_2_/F_I_O_2_ improvement ≥ 20% compared to baseline, %	NA	0	NA	NA	NA
	RR, bpm	23.8 (5.3)	21.3 (4.6)	NA	NA	0.461
	ROX index	6.5 (1.3)	8.3 (3.0)	NA	NA	0.068
	Comfort	7.8 (2.1)	8.0 (1.6)	NA	NA	0.317

PTIF, peak tidal inspiratory flow; SpO_2_, pulse oximetry; F_I_O_2_, fraction of inspired oxygen; RR, respiratory rate; ROX index = [(SpO_2_/F_I_O_2_)/RR]; comfort (0–10), 0 as the least and 10 as the most comfort; NA, not available

*p < 0.05 compared to baseline at matching flow

^†^*p* < 0.05 compared to 10 L/min above matching flow

^&^*p* < 0.05 compared to 20 L/min above matching flow

Using SpO_2_/F_I_O_2_ improvement ≥ 20% by increasing the flow above PTIF from the baseline to define responders, for the 30 patients whose PTIF was 20–30 L/min, only 8 (27%) patients had positive response with HFNC set 10 L/min above PTIF (meaning a set flow of 30–40 L/min), while 18 (60%) and 19 (63%) patients had positive response at 20 (meaning a set flow of 40–50 L/min) and 30 L/min (which represents a flow of 50–60 L/min) above PTIF, respectively. For the 14 patients whose PTIF was 40 L/min, only 1 (7%) and 4 (29%) patients met the positive response criteria with HFNC flow at 10 (set flow of 50 L/min) and 20 L/min (set flow of 60 L/min) above PTIF, respectively (Table 2).

#### Relationship between flow ratios and patient responses

**Fig. 1:**
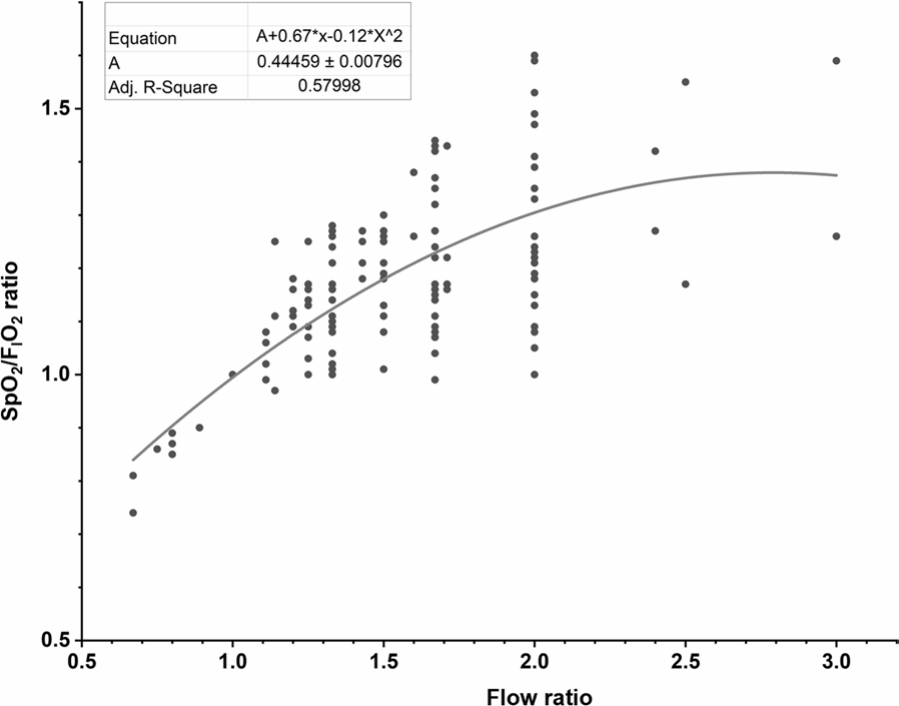
The correlation between SpO_2_/F_I_O_2_ ratio and flow ratio. The flow ratio of setting HFNC flow to the patient’s peak tidal inspiratory flow is shown on the X-axis. The ratio of patients’ SpO_2_/F_I_O_2_ at one flow setting to SpO_2_/F_I_O_2_ achieved when HFNC flow was set to match patient’s peak inspiratory flow during tidal breathing (matching flow) is shown on the Y-axis. The scatterplot shows significant correlation between the two ratios. SpO_2_, pulse oximetry; F_I_O_2_, fraction of inspired oxygen; HFNC, high-flow nasal cannula.

**Fig. 2:**
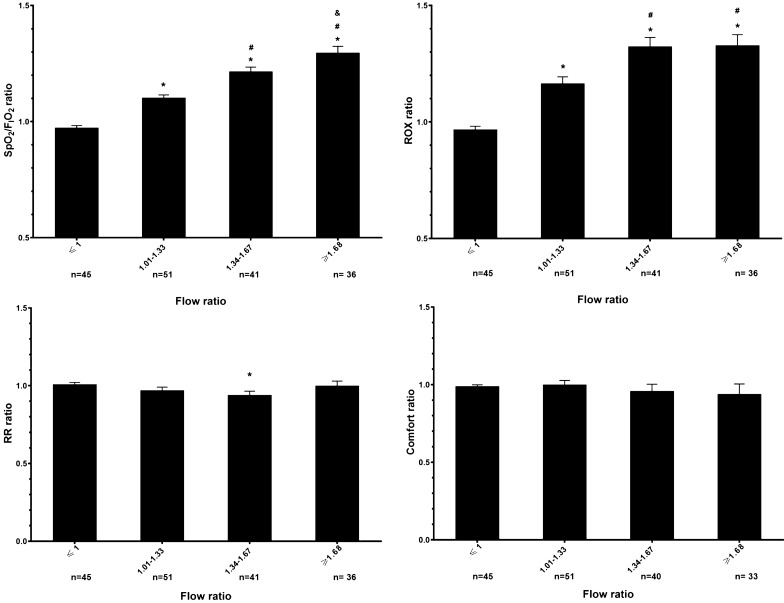
Patient response to different flow ratios. On the top 2 figures, X-axis is the flow ratio of setting HFNC flow to patients’ peak inspiratory flow during tidal breathing and the flow ratios are divided into four groups (≤ 1, 1.01–1.33, 1.34–1.67, and ≥ 1.68). On the Y-axis, the ratio of patients’ SpO_2_/F_I_O_2_ (left top) or ROX (right top) at the flow setting to SpO_2_/F_I_O_2_ or ROX at their matching flow are shown. As the flow ratio increased, the SpO_2_/F_I_O_2_ ratio increased. Similarly, compared to ROX ratio with flow ratios ≤ 1.33, ROX ratio was higher with flow ratios ≥ 1.34–1.67 and 1.68, but ROX ratio did not increase beyond flow ratios of 1.34–1.67. RR ratio was lower with flow ratios of 1.34–1.67 than with flow ratios ≤ 1 (left bottom). No significant differences of comfort score ratios were found at different flow ratios (right bottom). *p < 0.05 compared to flow ratios ≤ 1. ^#^*p* < 0.05 compared to flow ratios of 1.01–1.33. ^&^*p* < 0.05 compared to flow ratios of 1.34–1.67. SpO_2_, pulse oximetry; F_I_O_2_, fraction of inspired oxygen; RR, respiratory rate; ROX = (SpO_2_/F_I_O_2_)/RR

The ratio of SpO_2_/F_I_O_2_, defined as the value of SpO_2_/F_I_O_2_ at different gas flows to their SpO_2_/F_I_O_2_ at matching flow, increased as the flow ratio (defined as different gas flows to matching flow) increased (Fig. 1). By dividing the measured SpO_2_/F_I_O_2_ ratios into the different quartiles of the calculated flow ratios (≤ 1, 1.01–1.33, 1.34–1.67, and ≥ 1.68), we found that the SpO_2_/F_I_O_2_ ratio increased as flow ratios increased. Similarly, ROX index ratio (defined as the ROX index at different gas flows to their ROX at matching flow) increased as flow ratios increased, but no further improvement was observed with flow ratios of ≥ 1.68. RR ratio (defined as the RR at different gas flows to their RR at matching flow) significantly decreased at the flow ratios of 1.34–1.67. No significant differences were found in comfort scores among different flow ratios (Fig. 2).

### In vitro study

**Fig. 3:**
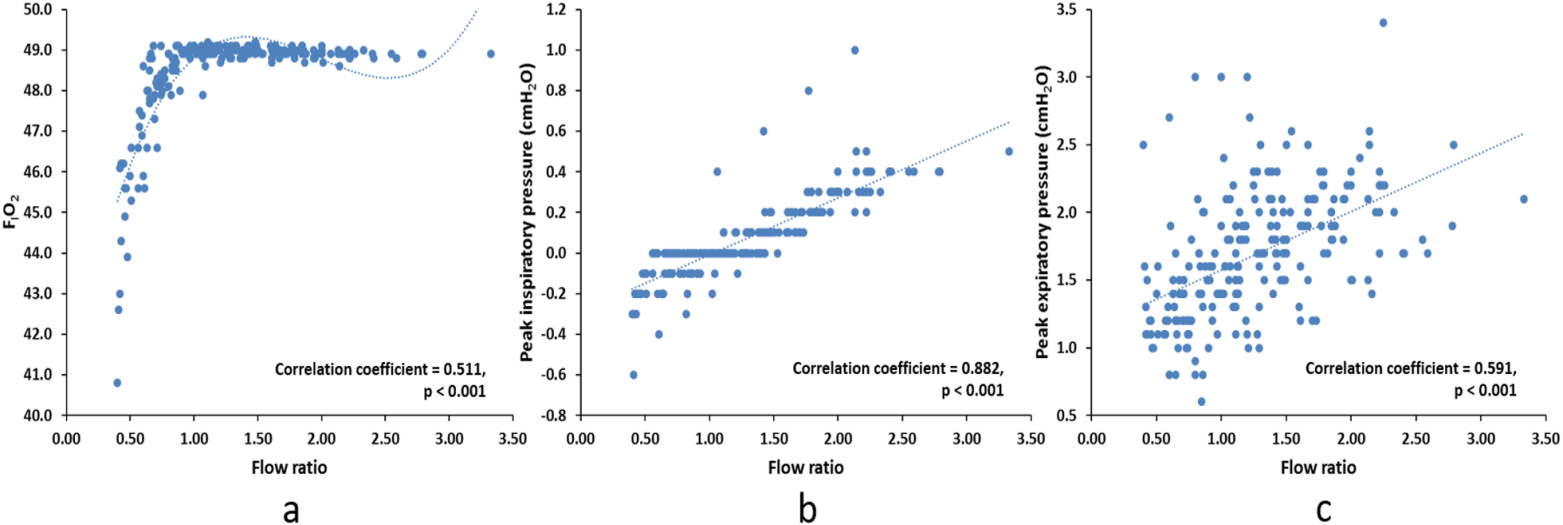
The correlation between flow ratio and F_I_O_2_, peak inspiratory and expiratory pressure at trachea in the in vitro study. X-axis is the flow ratio of setting HFNC flow to peak tidal inspiratory flow, Y-axis is the trachea F_I_O_2_ (left), peak inspiratory (middle) and peak expiratory pressure (right). As the flow ratio increased, both peak inspiratory and expiratory pressure increased, while F_I_O_2_ stabilized when flow ratio was ≥ 1. F_I_O_2_, fraction of inspired oxygen

In the in vitro study, we replicated the breathing patterns and HFNC gas flow settings of the 49 subjects enrolled in the clinical study. We found a significant correlation between the ratio of HFNC flow to PTIF (flow ratio) and tracheal F_I_O_2_ (*r* = 0.511, *p* < 0.001), tracheal peak inspiratory pressure (*r* = 0.882, *p* < 0.001) and peak expiratory pressure (*r* = 0.591, *p* < 0.001). Tracheal peak inspiratory and expiratory pressures increased as flow ratio increased, while F_I_O_2_ stabilized with flow ratios ≥ 1 (Fig. 3).

## Discussion

This study is the first to report the breathing patterns of non-intubated patients with AHRF. Patient oxygenation improved as HFNC flow increased up to two times of their PTIF in all patients. Interestingly, the results of the in vitro study suggested that the oxygenation improvement observed above the PTIF was more likely due to the increase in the airway pressure as F_I_O_2_ remained unchanged.

### Peak inspiratory flow during tidal breathing

Our findings showed that PTIF (34 [9] L/min) was higher in patients with AHRF compared to adult healthy volunteers (PTIF of 28 [9] L/min) [17] and was similar when compared to patients with tracheostomy (PTIF of 30 [27–32] L/min) [19]. Therefore, our findings align with the clinician assumption that patients with hypoxemia have higher PTIF than patients without hypoxemia [22]. However, we did not find a significant correlation between PTIF and the severity of hypoxemia.

In two recently published studies in intubated patients, PTIF varied from 25–65 L/min [20] to 40–80 L/min [21], values that were higher than PTIF in our patients. This might be explained by the need to overcome the resistance of an endotracheal tube. Butt and colleagues utilized the PTIF measured with intubation to guide HFNC flow settings after extubation. They found a significant correlation between the PTIF pre-extubation and the HFNC flow settings that patients had the greatest comfort after extubation [21]. Although PTIF measured during the spontaneous breathing trial while intubated slightly overestimated patient PTIF post-extubation, this strategy allows personalized flow titration immediately after extubation.

It should also be noted that, despite the obvious limitations in breathing pattern measurement, the breathing patterns described in the present study may provide a reference to establish settings for future in vitro studies that simulate spontaneous breathing of hypoxemic patients, such as studies on HFNC [15, 16, 18], noninvasive ventilation, or aerosol therapy [24].

### Patient responses to different HFNC flows

We observed oxygenation improvement as HFNC flow increased, which agrees with others’ observations [10, 12]. When HFNC flow was increased from 30 to 60 L/min in the study by Mauri and colleagues [10] or from 20 to 60 L/min in the study by Delorme and coworkers [12], both groups found significant improvement in oxygenation, lung aeration, dynamic compliance, and work of breathing. Based on their results, both groups of investigators recommended HFNC flow of 60 L/min for adult patients with AHRF [10, 12]. However, it is worth noting that there was a high heterogeneity in patient’s response to different flows and, therefore, individualizing flow settings during HFNC therapy seems to be a reasonable approach. It was also hypothesized that the oxygenation improvement observed may be due to the increase of oxygen delivery. However, our in vitro study showed that when the flow ratio was ≥ 1, tracheal peak inspiratory and peak expiratory pressures increased as flow ratio increased with no additional increase in the F_I_O_2_. Thus, these results suggest that the oxygenation improvement observed with flow that exceed the PTIF could be, at least in part, explained by the increased airway pressure generated by these higher flows. [16–18].

Respiratory rate decreased significantly at HFNC flows set at 1.34–1.67 times of PTIF and no further improvements in ROX index were found when HFNC flows were set at ≥ 1.68 times of PTIF. Similarly, Basile and co-investigators [13] set HFNC flow based on patient predicted body weight (PBW) for 12 patients with AHRF. According to their protocol of 0.5, 1.0, and 1.5 L/min/kg of PBW, they utilized median flows of 35, 65, and 100 L/min. They found that HFNC flow at 1.5 L/min/kg of PBW was worse tolerated and did not improve homogeneity of ventilation or increase in end-expiratory lung volume (EELV) compared with HFNC flow at 1.0 L/min/kg of PBW. Moreover, the change in ROX measured at 30 L/min and 60 L/min has been correlated with a change in EELV [25]. Importantly, in 30% of the patients, the ROX index and EELV decreased after increasing the flow. These findings support our observation that an arbitrary or maximum flow setting, such as 60 L/min, might exceed the individual plateau level in some patients but might be insufficient for other patients who have high PTIF. It should be noted that the increase in EELV and lung homogeneity associated at certain flows may reduce the likelihood of patient self-inflicted lung injury (P-SILI) [26]. This is noteworthy, as P-SILI may be associated with HFNC failure and need for mechanical ventilation. Thus, like for an intubated patient, personalizing the flow settings to minimize the risk of P-SILI may be a strategy to potentially improve outcomes in AHRF patients treated with HFNC.

Currently, there is no commercially available device to measure patient PTIF, and different HFNC flows may alter PTIF given that RR and inspiratory effort are affected by the flows used [11]. Therefore, it is unlikely that baseline PTIF could be used to optimize flow settings at the bedside during HFNC therapy. The present study highlights the reality that one size (or HFNC flow, in this case) does not necessarily fit all. Thus, a pragmatic solution to set HFNC flow would be to initiate HFNC at a flow of 40 L/min then rapidly titrate upwards based on response in ROX index and respiratory rate, as well as patient tolerance/comfort. Specifically, when the improvement in ROX index begins to plateau, then optimal HFNC flow has been achieved. It is worth noting that during this titration process, F_I_O_2_ needs to be adjusted to maintain a SpO_2_ between the target range (preferably 90–97%) at different HFNC flow settings [27, 28].

This study has certain limitations. First, the maximum studied flow was 60 L/min, and not all patients received HFNC flows of 20 and 30 L/min above their PTIF. Therefore, in patients whose PTIF was 40 L/min or higher, whether their responses to higher flow were the same as patients whose PTIF was 30 L/min or lower is unknown. Second, this was a short-term non-randomized study that might not reveal any long-term effects. Future studies are needed to understand the long-term benefits of the individualized HFNC flow settings with more frequent measurements and flow titration. Third, breathing pattern measurements were done while the patients were not using the HFNC device. However, we maintained the same oxygenation levels during the measurement, minimizing the effect that hypoxemia may have on the respiratory pattern. Fourth, we only assessed oxygenation, respiratory rate and patient comfort at different flows, which might not reflect the lung homogeneity during tidal ventilation. Similarly, we did not measure inspiratory effort and, therefore, significant improvements in terms of reducing P-SILI might be possible with higher flows despite no associated oxygenation improvement. Indeed, better oxygenation may not be necessarily related with better outcomes. Fifthly, the in vitro study was performed with the mouth closed, and a large-size cannula, thus, the pressures achieved might not reflect the ones during daily clinical practice. Finally, the effects on oxygenation were assessed using SpO_2_/F_I_O_2_ instead of partial pressure of oxygen (PaO_2_)/F_I_O_2_. That said, many AHRF patients treated by HFNC are monitored non-invasively and SpO_2_/F_I_O_2_ has been shown to be a convenient, noninvasive, and practical substitute for PaO_2_/F_I_O_2_ [27, 28]. Therefore, this noninvasive assessment on oxygenation is clinically useful and represents what is currently done in daily clinical practice during treatment with HFNC.

## Conclusion

Patients with AHRF present mean PTIF of 30–40 L/min, which did not increase with severity of hypoxemia. An increase in HFNC flows up to two times of the individual patient’s PTIF, incrementally improved oxygenation but the ROX index plateaued with HFNC flows of 1.34–1.67 times the individual PTIF. Oxygenation improvement observed with HFNC flow above the patient’s PTIF is largely due to the increase in airway pressures generated by higher flows. Thus, in patients with AHRF, setting the initial HFNC flow at 40 L/min with rapid incremental titration based on the improvement of oxygenation, respiratory rate, and patient tolerability, could be a pragmatic approach to optimize HFNC flows at the bedside.

## Supplementary Information

**Additional file 1: Figure S1**. The setup of device to measure patient breathing patterns. **Figure S2.** The in vitro experiment setup. **Figure S3.** Individual patient responses to different flow settings for patients whose PTIF is 20-30 L/min. **Figure S4.** Individual patient responses to different flow settings for patients whose PTIF is 40 L/min.

## Data Availability

De-identified data will be available upon reasonable request made by researchers with approved protocol after publication.

## Abbreviations

HFNC: High-flow nasal cannula
AHRF: Acute hypoxemic respiratory failure
PTIF: Peak tidal inspiratory flow
PEEP: Positive end-expiratory pressure
F_I_O_2_: Fraction of inspired oxygen
Vt: Tidal volume
Ti: Inspiratory time
RR: Respiratory rate
SpO_2_: Pulse oximetry
ECMO: Extracorporeal membrane oxygenation
ROX index: (SpO_2_/F_I_O_2_)/RR
SD: Standard deviation
IQR: Inter-quartile range
PBW: Predicted body weight
