# Supplementary material for: Optimizing high-flow nasal cannula flow settings in adult hypoxemic patients based on peak inspiratory flow during tidal breathing
Source: Ann Intensive Care. 2021 Nov 27;11:164. doi: 10.1186/s13613-021-00949-8 (PMC8626729; doi:10.1186/s13613-021-00949-8)
Supplement: Supplementary file 1 — Additional file 1: Figure S1. The setup of device to measure patient breathing patterns. Figure S2. The in vitro experiment setup. Figure S3. Individual patient responses to different flow settings for patients whose PTIF is 20-30 L/min. Figure S4. Individual patient responses to different flow settings for patients whose PTIF is 40 L/min. [file 13613_2021_949_MOESM1_ESM.pdf]

Optimizing High-Flow Nasal Cannula Flow Settings in Adult Hypoxemic Patients Based on Peak Inspiratory Flow during Tidal Breathing

Supplementary files

Fig. S1 The setup of device to measure patient breathing patterns p 2

Fig. S2 The in-vitro experiment setup p 3

Fig. S3 Individual patient responses to different flow settings for patients whose PTIF is 20-30 L/min p 4

Fig. S4 Individual patient responses to different flow settings for patients whose PTIF is 40 L/min p 5

Fig. S1 The setup of device to measure patient breathing patterns

A flow sensor was connected to a mask and Y-piece, with one end attached to a one-way valve for expiration, while the other end was attached to an oxygen reservoir bag with one-way valve for inspiration. Oxygen tubing from the reservoir bag was connected to a backpressure compensated flowmeter and an air-oxygen blender to provide inhaled gas with a constant F_IO_2. F_IO_2 was titrated to maintain SpO_2 at 90-97% during measurement. Flow sensor was connected to NICO2 monitor to measure patient's breathing profiles during tidal breathing, including peak tidal inspiratory flow, tidal volume, inspiratory time, and respiratory rate.

F_IO_2, fraction of inspired oxygen; SpO_2, pulse oximetry.

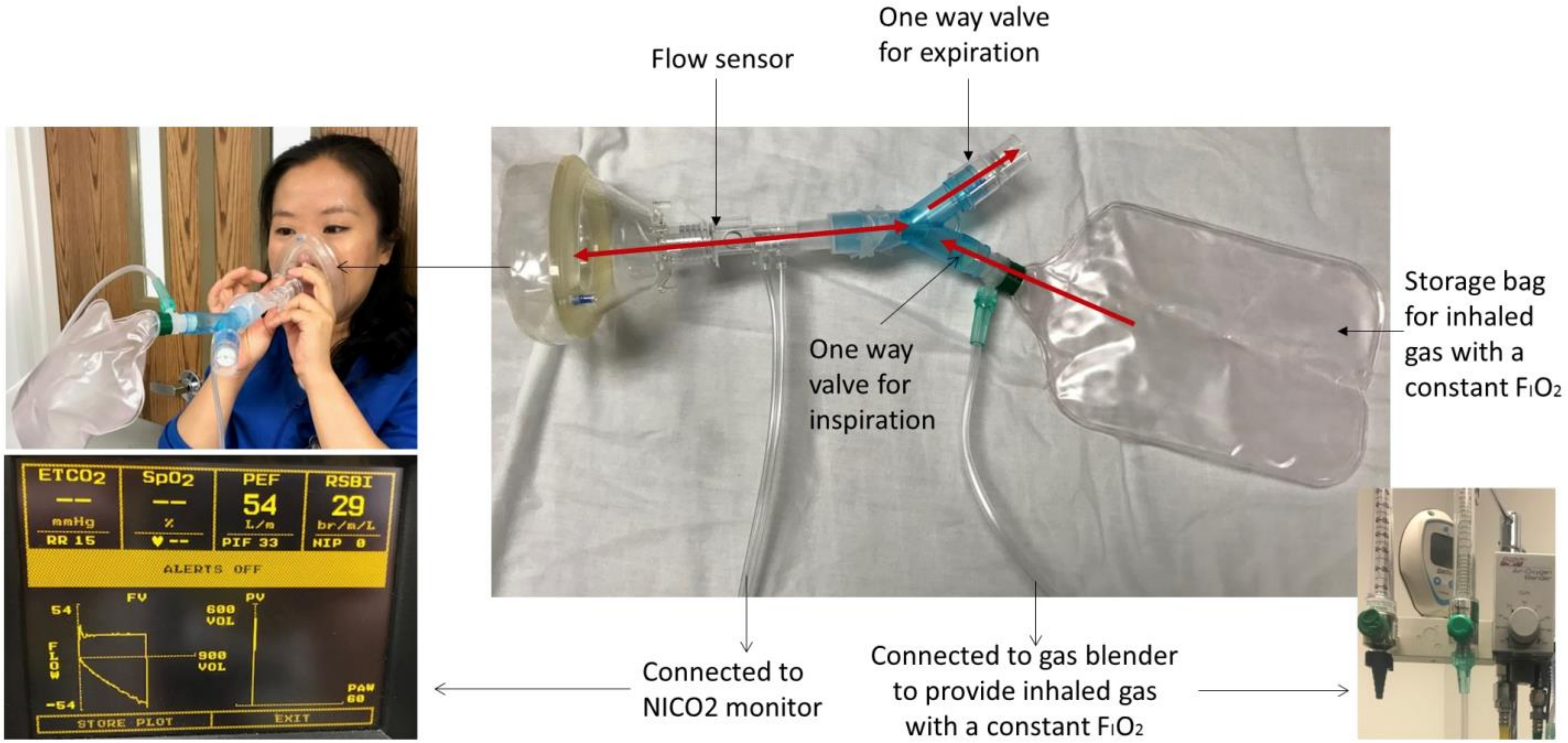

Fig. S2 The in-vitro experiment setup

An adult manikin (Laerdal adult airway management trainer, Stavanger, Norway) with size appropriate airway anatomy was attached to one chamber of a model lung (TTL, Michigan Instruments, Grand Rapids, USA), while the other chamber was connected to a critical care ventilator (Drager Evita XL, Drager, Lubeck, Germany) to simulate respiratory drive. The two chambers moved together via a rigid metal connector to simulate spontaneous breathing. Ventilator settings were adjusted to replicate the breathing patterns that were acquired from patients and the flow settings in the clinical study. A flow sensor and NICO2 monitor was connected between the manikin's trachea and the model lung to confirm the breathing patterns. Between the trachea and the model lung, a pressure manometer and an oxygen analyzer were connected via a T-piece to measure F_IO_2 and pressure, respectively at the trachea.

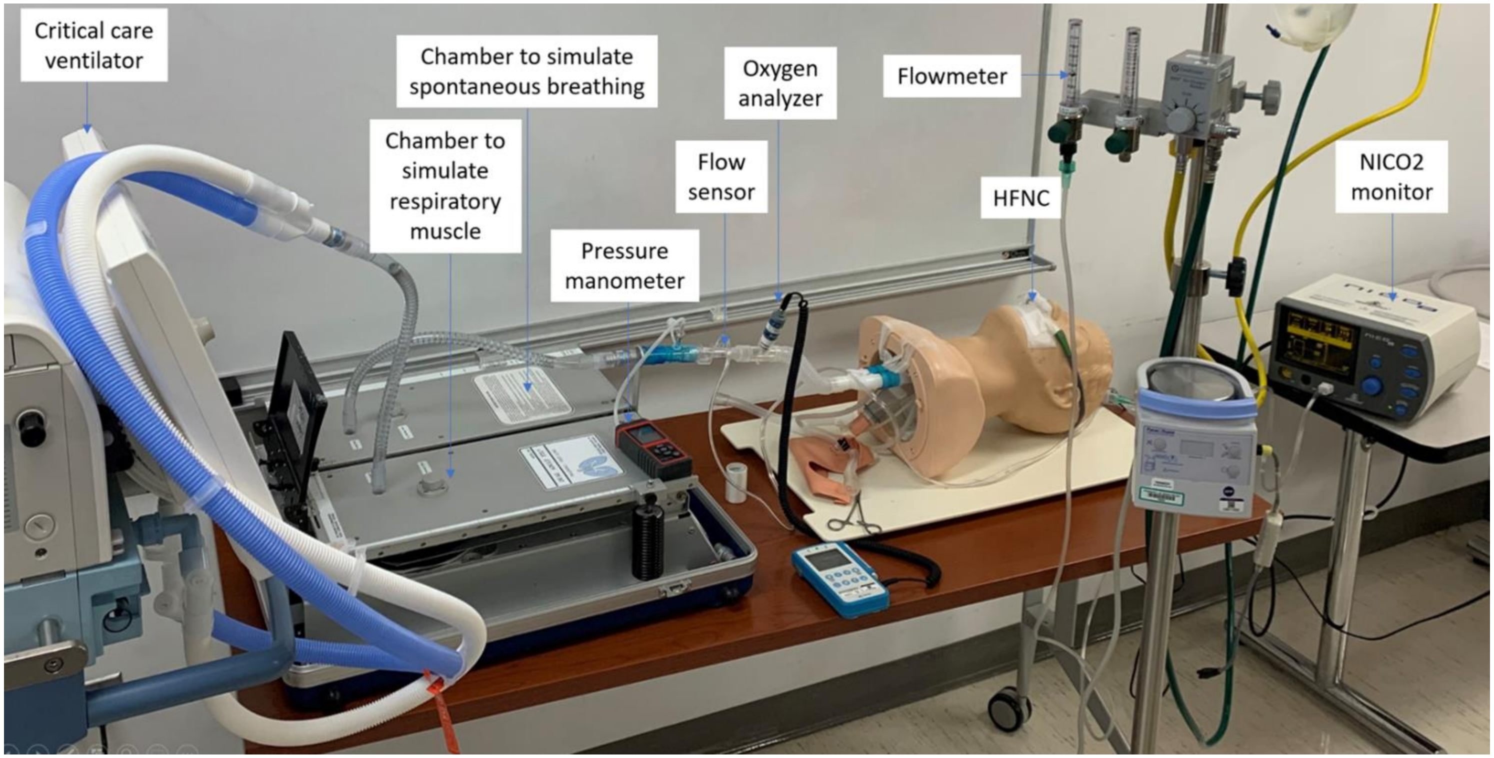

Fig. S3 Individual patient responses to different flow settings for patients whose PTIF is 20-30 L/min

For patients whose PTIF is 20-30 L/min, HFNC flow at 10 and 20 L/min above PTIF significantly improved SpO_2/F_IO_2 and ROX index, with comparison to HFNC flow at PTIF level. However, there was no significant differences of SpO_2/F_IO_2 and ROX index at 20 and 30 L/min above PTIF. PTIF, patient tidal inspiratory flow; SpO_2, pulse oximetry; F_IO_2, fraction of inspired oxygen; ROX, SpO_2/F_IO_2/respiratory rate.

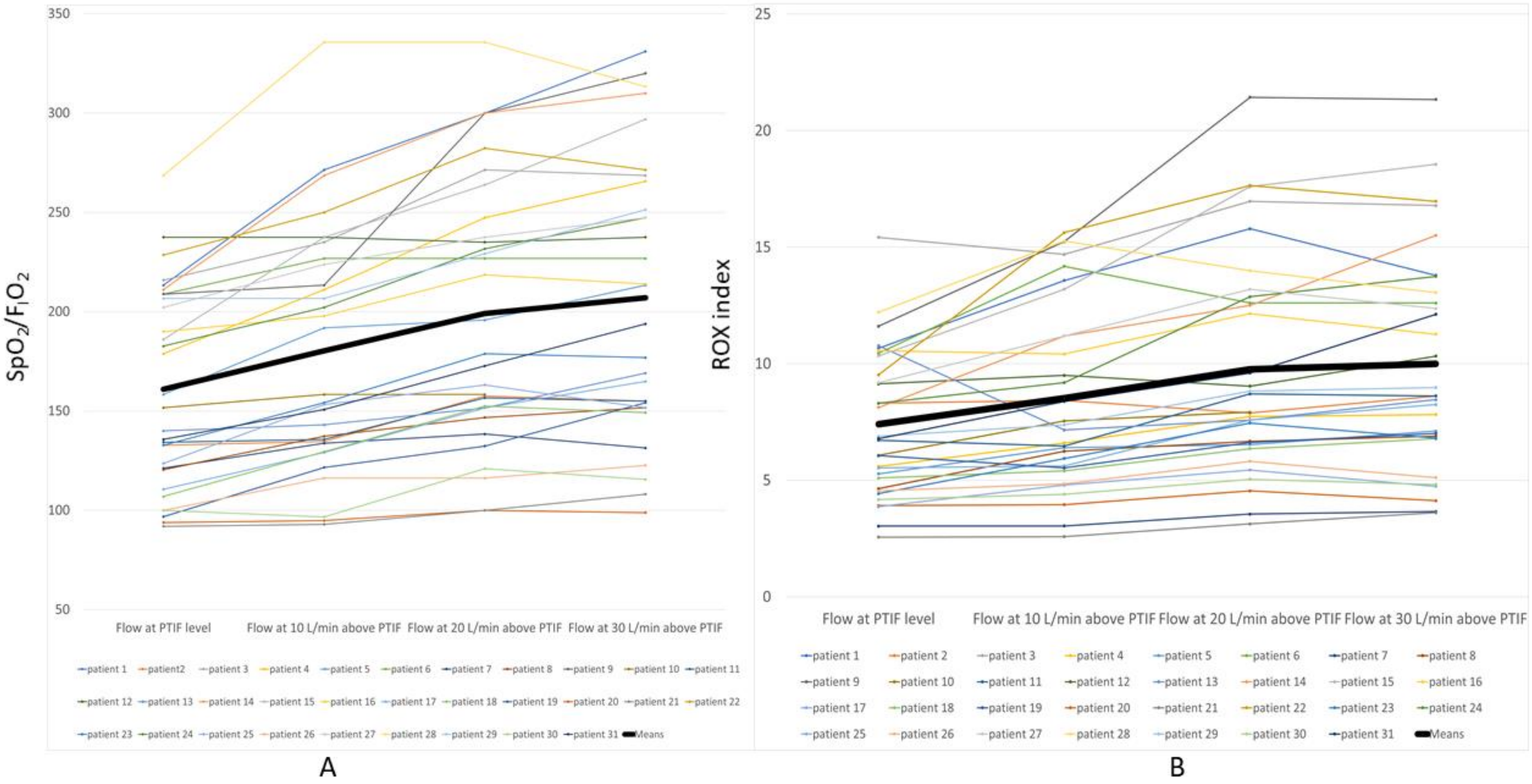

Fig. S4 Individual patient responses to different flow settings for patients whose PTIF is 40 L/min

For patients whose PTIF is 40 L/min, HFNC flow at 10 and 20 L/min above PTIF significantly improved SpO_2/F_IO_2 and ROX index, with comparison to HFNC flow at PTIF level. PTIF, patient tidal inspiratory flow; SpO_2, pulse oximetry; F_IO_2, fraction of inspired oxygen; ROX, SpO_2/F_IO_2/respiratory rate.

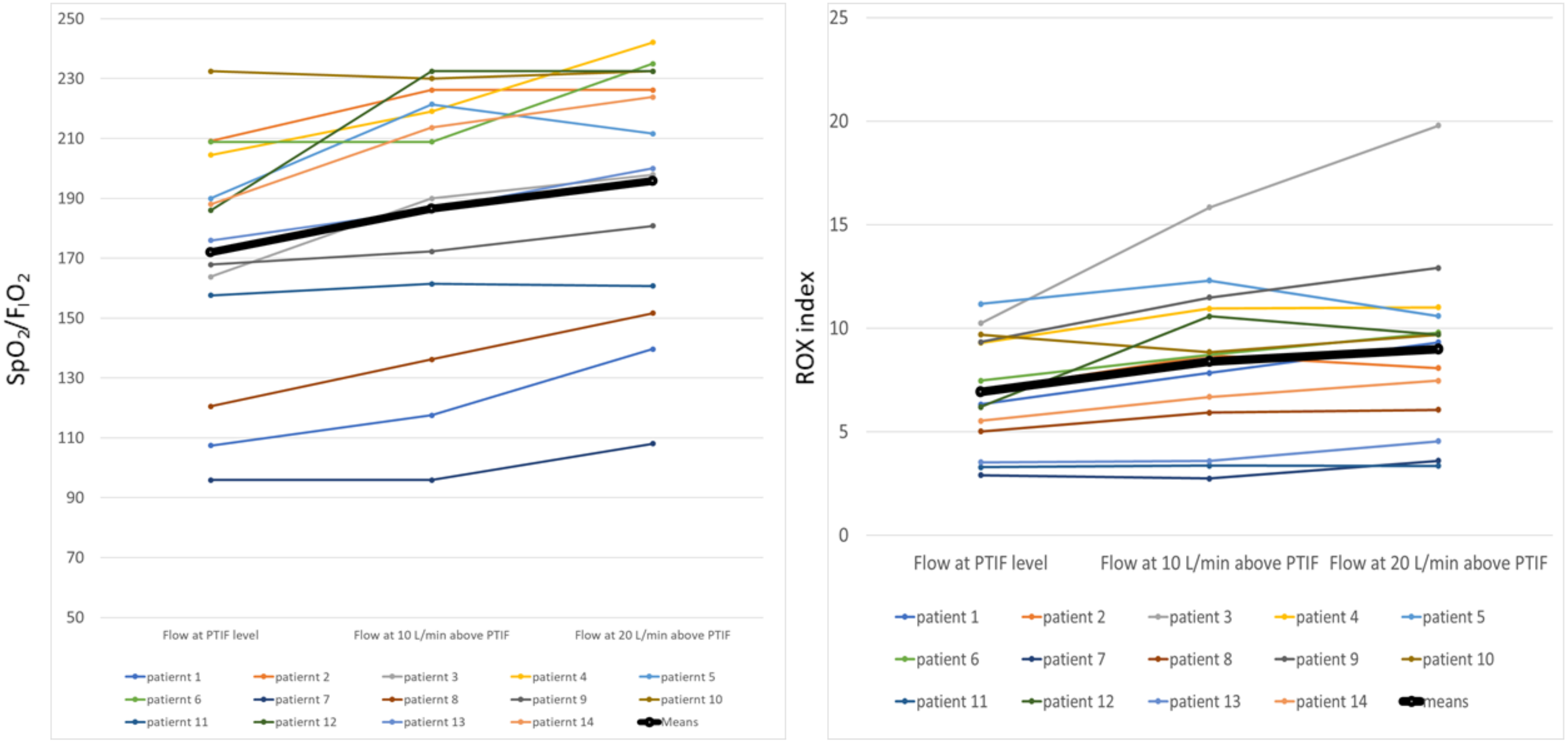

A B
